# Augmentation of VAMP-catalytic activity of botulinum neurotoxin serotype B does not result in increased potency in physiological systems

**DOI:** 10.1371/journal.pone.0185628

**Published:** 2017-10-05

**Authors:** Mark Elliott, Jacquie Maignel, Sai Man Liu, Christine Favre-Guilmard, Imran Mir, Paul Farrow, Fraser Hornby, Sandra Marlin, Shilpa Palan, Matthew Beard, Johannes Krupp

**Affiliations:** 1 Ipsen Bioinnovation, 102 Park Drive, Milton Park, Abingdon, United Kingdom; 2 Ipsen Innovation, Les Ulis, France; Institut Pasteur, FRANCE

## Abstract

Botulinum neurotoxins (BoNTs) are used extensively as therapeutic agents. Serotypes A and B are available as marketed products. Higher doses of BoNT/B are required to reach an efficacy similar to that of products containing BoNT/A. Advances in our understanding of BoNT/B mechanism of action have afforded the opportunity to make rational modifications to the toxin aimed at increasing its activity. Recently, a mutation in the light chain of BoNT/B (S201P) was described that increases the catalytic activity of the isolated BoNT/B light chain in biochemical assays. In this study, we have produced two full-length recombinant BoNT/B toxins in *E*.*coli*–one wild type (rBoNT/B1) and one incorporating the S201P mutation (rBoNT/B1_(S201P)_). We have compared the activity of these two molecules along with a native BoNT/B1 in biochemical cell-free assays and in several biological systems. In the cell-free assay, which measured light-chain activity alone, rBoNT/B1_(S201P)_ cleaved VAMP-2 and VAMP-1 substrate with an activity 3–4-fold higher than rBoNT/B1. However, despite the enhanced catalytic activity of rBoNT/B1_(S201P),_ there was no significant difference in potency between the two molecules in any of the *in vitro* cell-based assays, using either rodent spinal cord neurons or cortical neurons. Similarly in *ex vivo* tissue preparations rBoNT/B1_(S201P)_ was not significantly more potent than rBoNT/B1 at inhibiting either diaphragm or detrusor (bladder) muscle activity in C57BL/6N and CD1 mice. Finally, no differences between rBoNT/B1 and rBoNT/B1_(S201P)_ were observed in an *in vivo* digit abduction score (DAS) assay in C57BL/6N mice, either in efficacy or safety parameters. The lack of translation from the enhanced BoNT/B1_(S201P)_ catalytic activity to potency in complex biological systems suggests that the catalytic step is not the rate-limiting factor for BoNT/B to reach maximum efficacy. In order to augment the efficacy of BoNT/B in humans, strategies other than enhancing light chain activity may need to be considered.

## Introduction

Botulinum neurotoxins (BoNTs) are 150 kDa modular proteins consisting of two sub-units: an N-terminal light chain (LC) domain of 50 kDa and a heavy chain (HC) domain of 100 kDa, which are linked by a disulphide bond. The HC consists of two sub-domains: H_C,_ responsible for neuronal cell binding; and H_N_, a translocation domain which functions to deliver the LC from internalized endosomal compartments into the cytosol. The LC is a zinc-dependent metalloprotease that specifically cleaves soluble N-Ethylmaleimide-sensitive factor attachment receptor (SNARE) proteins–the drivers of synaptic vesicle fusion with the plasma membrane [1].

Seven serotypes of BoNT have been identified (termed A-G), which cleave different SNARE proteins at distinct residues: BoNT/A and BoNT/E cleave SNAP-25 (synaptosome-associated protein of 25 kDa), while BoNT/B, /D, /F and /G cleave VAMP-2 (vesicle associated membrane protein), and BoNT/C cleaves both SNAP-25 and syntaxin-1 [1]. SNARE protein cleavage by BoNT blocks synaptic vesicle fusion with the plasma membrane and neurotransmitter exocytosis from neuronal cells, leading to the flaccid paralysis characteristic of botulism.

Despite being the causative agent of botulism, BoNTs are extensively used as therapeutic agents in the clinic. Their principal use is in the treatment of disorders involving the hyperactivity of cholinergic fibres innervating skeletal muscle, such as cervical dystonia, spasticity, strabismus, and blepharospasm; though they are also used off-label in a wide variety of conditions [2]. Of the seven BoNT serotypes described, only BoNT/A and BoNT/B are commercially available as therapeutic products. The market is dominated by BoNT/A, mainly because in humans the efficacy of BoNT/A is greater than BoNT/B and consequently lower doses are required to achieve the desired therapeutic effect [3, 4]. In the treatment of muscular disorders, the clinically effective dose of BoNT/B is 40–50 times higher than that of BoNT/A when compared in equivalent mouse lethality units [4].

The therapeutic effects of BoNT are not permanent, which necessitates repeated injection of toxin. In a sub-set of patients, resistance to BoNT/A treatment is caused by the formation of blocking antibodies [5]. This is estimated to occur in approximately 1–3% of patients depending on indication and the assay method employed to detect the immune response [6, 7]. Use of BoNT/B is recommended in BoNT/A-resistant patients [8]; however, treatment with BoNT/B is associated with a higher incidence of immune response compared to BoNT/A [7]. This increased incidence of immunogenicity is most likely due to the increased protein load from the higher doses that are necessary to achieve therapeutic effect with BoNT/B [9].

Lowering the required treatment dose of BoNT/B may help to lower immune responses in patients. In order to achieve lower efficacious doses of BoNT/B, the toxin could be engineered to enhance its potency in humans. One way to achieve higher potency could be through mutations in the LC of BoNT/B (LC/B) to enhance its catalytic activity. The LC/B cleaves the vesicle associated VAMP-2 [10], ablating its ability to form a complex with SNAP-25 and Syntaxin-1 located on the plasma membrane. The molecular determinants of BoNT/B substrate recognition have been extensively studied [11–15], and this information has been utilised to engineer LC/B derivatives with elevated catalytic activity for VAMP-2 substrate cleavage [14, 15]. In one such derived LC/B molecule, the serine residue located within the S1′ binding pocket at position 201 was changed to proline (LC/B_(S201P)_). A proline residue is located in the corresponding position within the S1′ binding pocket of the LC of tetanus toxin (TeNT), a Clostridial toxin that cleaves VAMP-2 at the same scissile bond as BoNT/B. Using a recombinant LC/B_(S201P)_ in cell-free linear velocity reactions and in Neuro2A cell lysates, the S201P mutation increased the *K*_cat_ (substrate turnover) of LC/B approximately 10-fold, while the *K*_m_ (substrate affinity) was found to be unaltered [15].

In order to assess the therapeutic relevance of the S201P mutation, it is necessary to determine whether the incorporation of the LC/B_(S201P)_ derivative in a full-length BoNT/B1 toxin would result in a toxin with enhanced functional properties. We have produced a full-length BoNT/B1 toxin incorporating the S201P mutation recombinantly in *E*. *coli* (rBoNT/B1_(S201P)_). In this study, we describe the characterization of rBoNT/B1_(S201P)_ in a range of *in vitro*, *ex vivo* and *in vivo* assays, comparing its activity to wild type recombinant BoNT/B1 (rBoNT/B1) and a commercially sourced native BoNT/B1 purified from *C*.*botulinum*.

## Material and methods

### Cloning and expression of rBoNT/B1 and rBoNT/B1_(S201P)_ in *E*.*coli* and neurotoxin purification

The gene sequence encoding the botulinum neurotoxin serotype B1 (BoNT/B1; Okra strain) was subcloned into pET32a using standard molecular biology techniques. The S201P mutation was introduced by site directed mutagenesis of a single base (T601C) using appropriate oligonucleotides (5’-GGAATATGTGCCGGTTTTCAAC-3’ and the corresponding complementary strand). Each plasmid was transformed into BLR DE3 *Escherichia coli* for gene expression. Cells were grown at 37°C and 225 rpm shaking in 2 L baffled conical flasks containing 1 L modified Terrific Broth (mTB) supplemented with 0.1 g ampicillin. Once the A_600_ passed 0.5, the incubator temperature was decreased to 16°C, and the cells induced with 1 mM IPTG an hour later for 20 h. Cells were harvested at 8,000 rpm for 20 minutes and stored at -80°C until required.

Harvested cells were lysed by ultrasonication in 0.2 M NaCl, 50 mM Tris, pH 8 supplemented with Benzonase (Sigma), and clarified by centrifugation at 4500 rpm for 30 minutes at 4°C. The supernatant was passed through an anionic exchange column before adjusting to 1 M (NH_4_)_2_SO_4_ in 50 mM Tris, pH 8. The sample was further clarified by centrifugation and the target molecule purified by standard fast protein liquid chromatography (FPLC) techniques. This involved using a hydrophobic interaction resin (high performance butyl Sepharose, GE) for capture (1 M to 0.5 M (NH_4_)_2_SO_4_ in 50 mM Tris, pH 8 linear gradient) and an anion-exchange resin (high performance Q Sepharose, GE) for the intermediate purification step (0 M to 0.2 M NaCl in 50 mM Tris, pH 8 linear gradient). The partially purified molecule was then proteolytically cleaved with 0.2 μg/mL endoproteinase Lys-C for 2 h at 37°C to yield the active di-chain. This was adjusted to 1 M (NH_4_)_2_SO_4_ in 50 mM Tris, pH 8 and polished with a second hydrophobic interaction resin (high performance phenyl Sepharose, GE) to obtain the target molecule (1 M to 0.5 M (NH_4_)_2_SO_4_ in 50 mM Tris, pH 8 linear gradient). Each purified molecule was desalted into PBS pH 7.2, supplemented with 1 mg/mL BSA and stored at -80°C. Molecule identity was confirmed by SDS PAGE and Western blot analysis using a primary mouse anti-BoNT/B antibody against LC (in-house) and a primary chicken anti-BoNT/B antibody against H_C_ (#70–1003, Fitzgerald, US) and HRP-conjugated secondary antibodies (Sigma).

### Assessment of light chain protease activity (VAMP-2)

The protease activities of BoNT/B1 (List Laboratories, US), rBoNT/B1 and rBoNT/B1_(S201P)_ against VAMP-2 were assessed using the BoTest (Biosentinel, Wisconsin, US) cell-free assay. BoNT/B1, rBoNT/B1 and rBoNT/B1_(S201P)_ were diluted to 1.39 nM in BoTest Reaction Buffer (50 mM HEPES-NaOH, 5 mM NaCl, 10 μM ZnCl_2_, 0.1% Tween-20, 0.1 mg/mL BSA, pH 7.1). BoNTs were reduced at room temperature (20 ± 2°C) for 30 minutes by addition of 5 mM DTT to allow maximum catalytic activity in the assay. BoTest Reporter (VAMP-2 (33–94) flanked by N-terminal cyan fluorescent protein (CFP) and C-terminal yellow fluorescent protein (YFP) in 50 mM HEPES-NaOH, 10 mM NaCl, 15% glycerol) at a final concentration of 200 nM was combined with reduced BoNT/B1, rBoNT/B1 or rBoNT/B1_(S201P)_ (final concentration 0.5 pM—1.25 nM) in black Maxisorp plates (Nunc) in a final assay volume of 100 μL/well. The plates were sealed, wrapped in aluminium foil to prevent degradation of the light-sensitive substrate and incubated at 30°C for 18 h. After incubation, fluorescence emission at 485/20 nm and 528/20 nm following excitation at 440/40 nm was measured using a BioTek Synergy HT plate reader.

### Assessment of light chain protease activity (VAMP-1)

A plasmid encoding glutathione-S-transferase (GST) tagged VAMP-1 (2–96)-GFP was transformed into BL21 *E*. *coli* cells. Cells were grown at 37°C in 3 L mTB supplemented with 0.1 g ampicillin until the A_600_ had passed 0.5, after which the temperature was decreased to 16°C, and the cells induced with 1 mM IPTG. After 24 hr, cells were harvested at 9,000 x *g* for 20 minutes and lysed at 20 kpsi in a homogeniser (Constant Systems, UK) in 50 mM Hepes, 0.2 M NaCl, 5 mM DTT, pH 7.2 supplemented with benzonase. GST-tagged VAMP-1 (2–96)-GFP was purified from the clarified lysate using a GSTrap 4B Sepharose HP column (GE). The GST tag was removed by proteolytic cleavage by PreScission protease (Sigma, UK) before passing through a second GSTrap 4B Sepharose HP column to capture GST. Purified VAMP-1 (2–96)-GFP was concentrated and stored at -80°C.

VAMP-1 (2–96)-GFP in 50 mM Tris-HCl, 20 mM NaCl, 10 μM ZnCl_2_, pH 7.2 at a final concentration of 2 μM was combined with BoNT/B1, rBoNT/B1 or rBoNT/B1_(S201P)_ (final concentration 0.1 pM—10 nM) in a microtitre plate in triplicate (final assay volume of 20 μL/well). The plates were sealed, and incubated at 37°C for 24 h. After incubation, 20 μL 2x NuPAGE LDS sample buffer (Life Technologies, UK) and DTT (final concentration 10 mM) was added to each well and the plate was heated at 70°C for 5 minutes. Samples were separated by SDS-PAGE on 12% Bis/Tris gels for 50 minutes at 200 V. Gels were stained with Simply Blue Safe Stain (Life Technologies, UK), de-stained and imaged using a PXi 6 Access (Syngene, UK). Densitometry of bands for full-length and cleaved substrate was performed to estimate the percentage of VAMP-1 cleavage.

### Rat spinal cord neuron culture and [^3^H]-glycine release assay

Rat spinal cord neurons were prepared from E15 Sprague-Dawley rat embryos (Charles River, Margate, UK). Pregnant female rats were killed by CO_2_ asphyxiation under Schedule 1 of the Animals (Scientific Procedures) Act UK 1986 and the embryos were removed. Dissected spinal cords were dissociated in trypsin-EDTA for 45 minutes at 37°C, then triturated to a single cell suspension in plating medium (minimal essential medium (MEM) containing 2 mM GlutaMAX, 5% horse serum, 0.6% D-glucose and 0.15% NaHCO_3_). Cells were plated in matrigel coated 96-well plates at a density of 125,000 cells/well in 125 μL plating medium. Cells were maintained at 37°C in a humidified atmosphere containing 10% CO_2_. 24 hours after plating, medium was replaced with 125 μL MEM containing 2 mM GlutaMAX, 5% horse serum, 0.6% D-glucose, 0.15% NaHCO_3_, 2% N2 supplement, 40 ng/mL corticosterone and 20 ng/mL triiodothyronine. On DIV (days *in vitro*) 6, 60 μM 5-fluoro-2’-deoxyuridine (FdU) and 145 μM uridine were added to the medium to prevent proliferation of non-neuronal cells. The medium in each well was doubled to 250 μL on DIV 8. Cells were maintained by replacement of half the medium every 3–4 days.

Glycine release was assessed in spinal cord neurons at DIV 20–23. Spinal cord neurons were treated with a concentration-range of BoNT/B1, rBoNT/B1 or rBoNT/B1_(S201P)_ (30 fM—3 nM) for 24 hours at 37°C. Following removal of neurotoxin, cells were briefly washed twice in HEPES-buffered salt solution (HBS) (136 mM NaCl, 3 mM KCl, 2 mM CaCl_2_, 1 mM MgCl_2_, 10 mM HEPES, 10 mM glucose, pH 7.2). Cells were loaded with 2 μCi/mL [^3^H]-glycine (Perkin Elmer, UK) in HBS for 60 minutes at 35°C. Following removal of [^3^H]-glycine, cells were briefly washed 3 times with HBS. Basal and stimulated [^3^H]-glycine release were established by incubation at 35°C for 5 minutes with 50 μL/well assay medium containing low potassium (3 mM KCl), or high potassium (60 mM KCl) HBS solutions, respectively. To determine retained [^3^H]-glycine, cells were lysed by adding 50 μL/well RIPA buffer (Sigma, UK). Superfusates and cell lysates were transferred into 96-well Isoplates (Perkin Elmer, UK) and 200 μL/well OptiPhase Supermix scintillation fluid was added. Radioactivity was quantified using a MicroBeta2 plate reader (Perkin Elmer, UK).

### Rat cortical neuron culture and glutamate release assay

Rat cortical neurons were prepared from E17-E18 CD rat embryos (Charles River, Margate, UK). Pregnant female rats were killed by CO_2_ asphyxiation under Schedule 1 of the Animals (Scientific Procedures) Act UK 1986 and the embryos were removed. Dissected cortical tissue was collected into ice-cold Hank’s Balanced Salt Solution (HBSS) without Ca^2+^ or Mg^2+^, and then dissociated in papain solution for 40 minutes at 37°C following the manufacturer’s instructions (Worthington Biochemical, US). Cortical cells were plated on poly-L-ornithine (PLO) coated 96-well plates at a density of 20,000 cells/well in 125 μL Neurobasal media containing 2% B27 supplement, 0.5 mM GlutaMAX, 1% foetal bovine serum (FBS) and 100 U/mL penicillin/streptomycin. Cells were maintained at 37°C in a humidified atmosphere containing 5% CO_2_. A further 125 μL Neurobasal medium containing 2% B27, 0.5 mM GlutaMAX was added on DIV 4. Cells were maintained by replacement of half the medium every 3–4 days. On DIV 11, 1.5 μM cytosine β-D-arabinofuranoside (AraC) was added to the medium to prevent proliferation of non-neuronal cells.

Glutamate release was assessed in cortical neurons at DIV 19–21. Cortical neurons were treated with a concentration-range of BoNT/B1, rBoNT/B1 or rBoNT/B1_(S201P)_ (30 fM—3 nM) for 24 hours or rBoNT/B1 or rBoNT/B1_(S201P)_ (3 pM—30 nM) for 5 hours at 37°C. Following removal of neurotoxin, cells were briefly washed twice in Neurobasal medium containing 2% B27, 0.5 mM GlutaMAX and then pre-incubated in assay medium (Neurobasal w/o phenol red, 2% B27, 0.5 mM GlutaMAX, 10 μM TFB-TBOA ((3S)-3-[[3-[[4-(Trifluoromethyl)benzoyl]amino]phenyl]methoxy]-L-aspartic acid, excitatory amino acid transporter inhibitor, Tocris, UK) on a heat block at 35°C for 30 minutes. Following pre-incubation, cells were briefly washed once in assay medium. Basal and stimulated glutamate release were established by incubation at 35°C for 5 minutes with 40 μL/well assay medium containing low potassium (5 mM KCl), or high potassium (60 mM KCl), respectively. Cell superfusates were collected and glutamate content assessed using an Amplex Red glutamic acid assay (Invitrogen, UK). 10 μL superfusates were combined with 10 μL detection reagent (100 mM Tris-HCl, pH 7.4 containing 26 μg/mL Amplex UltraRed, 0.25 U/mL horseradish peroxidase, 0.08 U/mL glutamate oxidase, 0.5 U/mL glutamate pyruvate transaminase and 200 μM alanine) in black 384-well Optiplates (Perkin Elmer). Plates were incubated for 30 minutes at 37°C after which 5 μL Amplex Red Stop reagent (Life Technologies, UK) was added to each well. Fluorescence emission at 590 nm following excitation at 535 nm was determined using an Envision plate reader (Perkin Elmer). Glutamate concentrations of superfusates were determined by interpolation from a glutamate standard curve also run in each assay.

### Cortical neuron patch clamp electrophysiology

Rat cortical neurons were plated onto PLO-coated glass coverslips in 24-well plates at a density of 200,000 cells/well, prepared and maintained as above. Cortical neurons were treated with a concentration-range of BoNT/B1, rBoNT/B1 or rBoNT/B1_(S201P)_ (1 pM—10 nM) for 24 hours. Following removal of neurotoxin, cells were briefly washed twice in Neurobasal medium containing 2% B27, 0.5 mM GlutaMAX. Whole-cell recordings were performed in cortical neurons at DIV 19–21 at 35°C using pipettes pulled from borosilicate glass capillaries (Sutter) with a resistance of 4–7 MOhm when filled with the following solution: 144.8 mM K-gluconate, 0.1 mM CaCl_2_, 1 mM MgCl_2_, 10 mM HEPES, 0.1 mM NaGTP, 0.2 mM MgATP, 5 mM glutathione, 1 mM EGTA (pH 7.4, adjusted with KOH). Liquid junction potentials were not corrected. Cultures were continuously perfused with artificial cerebrospinal fluid (aCSF) (140 mM NaCl, 1.25 mM NaH_2_PO_4_, 3.5 mM KCl, 2 mM CaCl_2_, 1 mM MgCl_2_, 13 mM glucose; bubbled with 95% O_2_/5% CO_2_ (pH 7.4). Mini excitatory post-synaptic currents (mEPSCs) were recorded at a holding potential of -70 mV and continuously perfused with tetrodotoxin (TTX; 500 nM) to block sodium channels, bicuculline (10 μM) to block GABA_A_ receptors and CGP55845 (1 μM) to block GABA_B_ receptors. Signals were recorded using a Multiclamp 700B (Axon instruments) and sampled at a frequency of 20 kHz (filtered to 10 kHz) using a Digidata 1550A (Axon instruments). Recorded traces were filtered further using Clampex software to a frequency of 1 kHz using an online bessel filter.

### Animals for *ex vivo* and *in vivo* experiments

Work on C57BL/6N mice was carried out in laboratories in France, whereas work on CD1 mice was carried out in laboratories in the UK. France: All studies were approved by the ethical committee of Ipsen Innovation and performed in full compliance with the European Communities Council Directive (2010/63/EU of September, 22nd, 2010 on the protection of animals used for scientific purpose) and the French national Committee for the care and use of laboratory animals (Decree n° 2013–118). UK: Animals were killed under Schedule 1 of the Animals (Scientific Procedures) Act 1986. No approval from an ethics committee was sought for the use of animals and acquisition of embryos in this study.

For *ex vivo* experiments on C57BL/6N mice, female animals weighing 18-25g were purchased from Charles River (Lyon, France) or Janvier (Le Genest-Saint-Isle, France) and allowed to acclimatize for at least 5 days before the experiments. CD1 mice were purchased from Charles River Laboratories, Margate, UK. The animals were used for *ex vivo* experiments on the day of arrival. For *in vivo* experiments, female C57Bl/6N mice weighing 18-20g were purchased from Janvier labs (Le Genest-Saint-Isle, France). Animals were housed in groups of six per cage and acclimatized for 7 days before the beginning of the experiments. The mice had free access to water and were fed with pelleted complete diet *ad libitum*.

### Mouse phrenic nerve hemi-diaphragm *ex vivo* organ bath assay

For mouse hemi-diaphragm and bladder studies, neurotoxin dilutions were prepared as needed, in Krebs-Henseleit buffer (KHB; 118 mM NaCl, 4.7 mM KCl, 2.5 mM CaCl_2_, 1.2 mM KH_2_PO_4_, 1.2 mM MgSO_4_, 25 mM NaHCO_3_, 11 mM glucose, pH 7.5) supplemented with 0.5% (w/v) gelatin type A.

On the day of experiment, C57BL/6N mice were anaesthetised with isoflurane (3.5% in 2% O_2_) and exsanguinated before tissue collection; CD1 mice were killed by CO_2_ asphyxiation. The left phrenic nerve and hemi-diaphragm were removed from the thoracic cage. The muscles were fixed onto a custom electrode tissue holder and suspended (1 g passive tension) in organ baths (Emkabath4, Emka Technologies, Paris, France) containing 10 mL of KHB at 37°C and gassed with carbogen (95% O_2_/5% CO_2_). The phrenic nerve was continuously electro-stimulated with 20 μsec pulses delivered at 1 Hz. Contraction force was measured with isometric transducers (Emka Technologies). The preparations were allowed to equilibrate for 45 minutes in medium renewed every 15 minutes. Following equilibration, each tissue was subjected to a maximal concentration of tubocurarine (3 μM). The full inhibition of the signal was considered as an indication that the contractile response was mainly due to acetylcholine released by nerve stimulation (preparations with inhibition lower than 95% were discarded). The preparations were then washed extensively. After a washout period of 20–30 minutes, when the contractile response was stable, BoNT/B1, rBoNT/B1 or rBoNT/B1_(S201P)_ were added to the bath at a final concentration of 10 pM. Experimental data were recorded with software IOX v2.9 from Emka Technologies.

The decrease in contraction force of the hemi-diaphragm muscle following toxin addition was calculated as a percentage of the contraction immediately prior to toxin addition and a four-parameter logistic curve fitted to the data using GraphPad Prism (version 6.07, GraphPad Software, La Jolla California USA). From the curve fitted to the data, the times to 10% (t_10_) and 50% (t_50_) diaphragm paralysis were estimated.

### Mouse bladder strip *ex vivo* organ bath assay

On the day of experiment, mice were anaesthetised as described above. Mouse bladders were collected and cleaned of surrounding adipose and connective tissue. After the dome and base of the organ were removed, the urothelium and most of the lamina propria were dissected to leave the detrusor muscle. Two strips measuring about 6 x 2 mm were cut out from one bladder, fixed onto a custom electrode tissue holder and tensed to 0.5 g in organ baths (Emkabath4, Emka Technologies, Paris, France) filled with KHB and bubbled with carbogen at 37°C, pH 7.4. Bladder contraction force was measured with isometric transducers (Emka Technologies). After an equilibration period of approximately 45 minutes, with renewal of the buffer every 15 minutes, 70 mM KCl was applied to check the smooth muscle integrity after the dissection. After several washes and return to baseline, 10 μM carbachol was used to test post-synaptic muscarinic receptors. Subsequently, contractions of the detrusor muscle were evoked by electrical field stimulation using trains of 20 pulses (intra-train frequency 10 Hz, single pulse duration 20 μs) separated by 1 minute intervals. This protocol was specifically designed to depolarize autonomic nerves in the mouse detrusor plexus, and to generate release of cholinergic and non-noradrenergic, non-cholinergic (NANC) transmitters (ATP, Substance P, etc.) which could be quantitatively inhibited by BoNTs [16, 17].

The model had been validated beforehand by adding 1 μM tetrodotoxin (voltage-gated sodium channel blocker) into the bath to check that the contractions were of neurogenic origin. 1 μM atropine (muscarinic receptor antagonist) and 10 μM α-β-methylene-ATP (P2X receptor desensitizer) were able to completely inhibit the signal, indicating the co-release of acetylcholine and ATP from the parasympathetic nerve endings in the detrusor plexus [18].

After intensive washes and after at least 40 minutes of stable contractions, BoNT/B1, rBoNT/B1, or rBoNT/B1_(S201P)_ was added to the baths, at a final concentration of 1 nM. The amplitude of contractions was measured until 90% of the signal was abolished. At the end of the experiment a final addition of 10 μM carbachol was performed to assess the viability of the tissue. Experiments were rejected when post-stimulation carbachol responses were less than 80% of the pre-stimulation carbachol response. The times to 10% (t_10_) and 50% (t_50_) muscle paralysis were estimated in the same way described for the mouse phrenic nerve hemi-diaphragm assay.

### Mouse Digit Abduction Score (DAS) assay

The Digit Abduction Score (DAS) was used to determine local muscle weakening efficacy. The mice were suspended briefly by the tail to elicit a characteristic startle response, in which the animals extend their hind limbs and abduct their hind digits [19, 20]. This reflex is inhibited by administration of BoNT into the gastrocnemius-soleus muscle complex of the hind paw. Following BoNT injections at specified intervals, the varying degrees of digit abductions are scored on a five-point scale: from DAS 0 = normal (no muscle weakening) to DAS 4 = maximal reduction in digit abduction and leg extension (maximal muscle weakness).

Prior to the experiment, mice presenting an abnormal paw or a DAS value different from 0 were excluded. Mice were identified by tail markings. Each dose was tested on 6 mice. Experimenters were blinded to the toxin treatments.

Mice were anaesthetized with 4% isoflurane in oxygen. Each mouse received an intramuscular injection of BoNT/B1 (0.25–5.7 pg/mouse), rBoNT/B1, rBoNT/B1_(S201P)_ (0.3–5.2 pg/mouse), or vehicle in gelatin phosphate buffer (GPB: Na_2_HPO_4_ (Merck) 5mM plus gelatin 10% (M/V) (Sigma) to a final concentration of 2%, pH adjusted to 6.5 with orthophosphoric acid (Merck)) in the gastrocnemius-soleus muscle complex of the right hind paw. Injections were made with a fixed volume of 20 μL using a 30 gauge needle attached to a 100 μL glass syringe (s.g.e.). The needle was inserted under the skin 2 mm anterior to the gastrocnemius muscle and pushed into the middle of the muscle.

DAS measurements were performed daily for the first 4 days after injection. For analysis, the highest average DAS score per dose group during this period was plotted and fit by a nonlinear equation (Eq 1), with Lower DAS limit set to 0 and Upper DAS limit set to 4.

$$Mean\;DAS=Lower\;DAS\;limit+\frac{Upper\;DAS\;limit}{1+exp(-slope(log(Dose)-log(ED50)))}$$ (Eq 1)

ED_50_ values and their lower and upper 95% confidence intervals (C.I.) were derived from these fits. DAS 4 values are given as the first tested dose where all animals within that group were scored as DAS 4.

In parallel to the DAS measurements, mice were weighed daily. The variation of the body weights (BW) after treatments was calculated from the baseline body weight (before injections) for each mouse then averaged in groups. The percentage variation average BW of each dose group was statistically compared daily to the percentage variation average BW of the vehicle control group. The dose inducing BW loss of 10% (BW-10%) was calculated using the linear regression equation of the percentage variation BW around -10% corresponding to three doses. The Maximal Tolerated Index (MTI) was subsequently calculated as the ratio of BW-10% and ED_50_. At the end of the experiments, mice were euthanized by CO_2_ asphyxiation according to the method described in the directive 2010/63/EU.

### Data handling and statistical analysis

All data are expressed as individual data or as mean ± standard error of the mean (s.e.m.) of *n* independent experiments. All concentration-effect data were fitted to a four-parameter logistic equation (Eq 2):

$$Y=\frac{Bottom+(top-bottom)}{1+exp(\log(IC50-X)\;*slope}$$ (Eq 2)

For statistical analysis, significant differences between BoNT/B1, rBoNT/B1 and rBoNT/B1_(S201P)_ were determined using a one-way ANOVA with Dunnett’s *post-hoc* test, where appropriate. All data processing and statistical tests were performed using GraphPad Prism software (version 6.07, GraphPad Software, La Jolla California USA).

## Results

### Expression and purification of rBoNT/B1 and rBoNT/B1_(S201P)_

**Fig 1:**
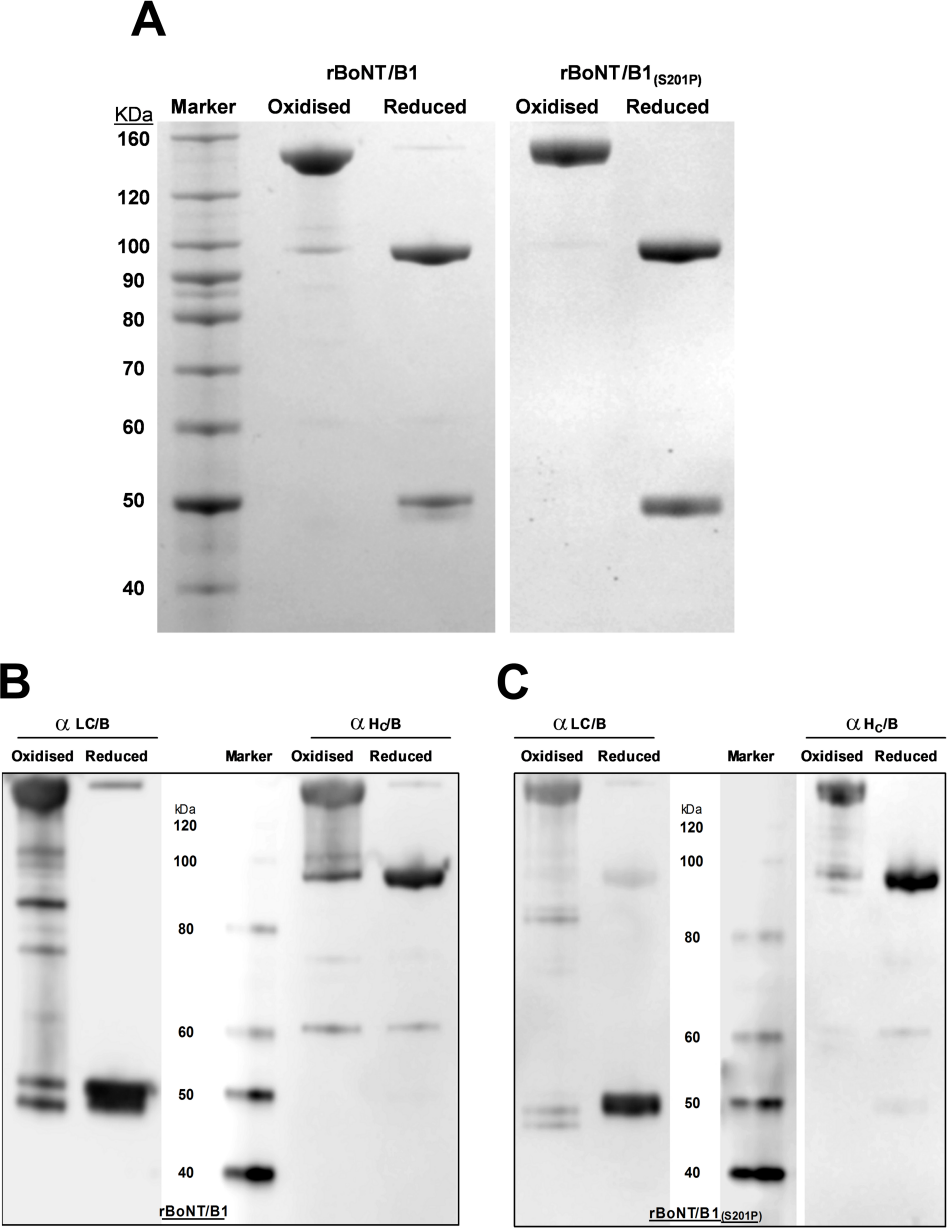
Purification of rBoNT/B1 and rBoNT/B1_(S201P)_. (A) The wild-type molecule, rBoNT/B1 (*left)* and single mutant, rBoNT/B1_(S201P)_ (*right*) were purified using a four step process–an initial passage through an anionic exchange column (AEC) before capture by hydrophobic interaction chromatography (HIC), followed by an intermediate AEC purification step and final HIC polish step after proteolytic cleavage into the active di-chain. Purified samples were resolved by PAGE and visualised with SafeStain. Western blot analysis of rBoNT/B1 (B) and rBoNT/B1_(S201P)_ (C) confirm the presence of the parent di-chain at ~150 kDa (“oxidised”) and light and heavy chains at ~50 kDa and ~100 kDa, respectively (“reduced”).

Both wild-type and mutant rBoNT/B constructs were derived from the BoNT/B1 sub-serotype (Okra strain) and purified using four chromatographic steps–involving two anionic exchange resins and two hydrophobic interaction resins. The single chain molecule was treated with endoproteinase Lys-C to yield the active di-chain. Purified product and confirmation of cleavage was assessed by SDS PAGE and Western blot analysis (Fig 1). The purities of rBoNT/B1 and rBoNT/B1_(S201P)_ were 89% and 83%, respectively. rBoNT/B1 was determined to be 98% activated, rBoNT/B1_(S201P)_ was 100% activated.

### Cell-free cleavage of VAMP-2 and VAMP-1 by BoNT/B1, rBoNT/B1 and rBoNT/B1_(S201P)_

**Fig 2:**
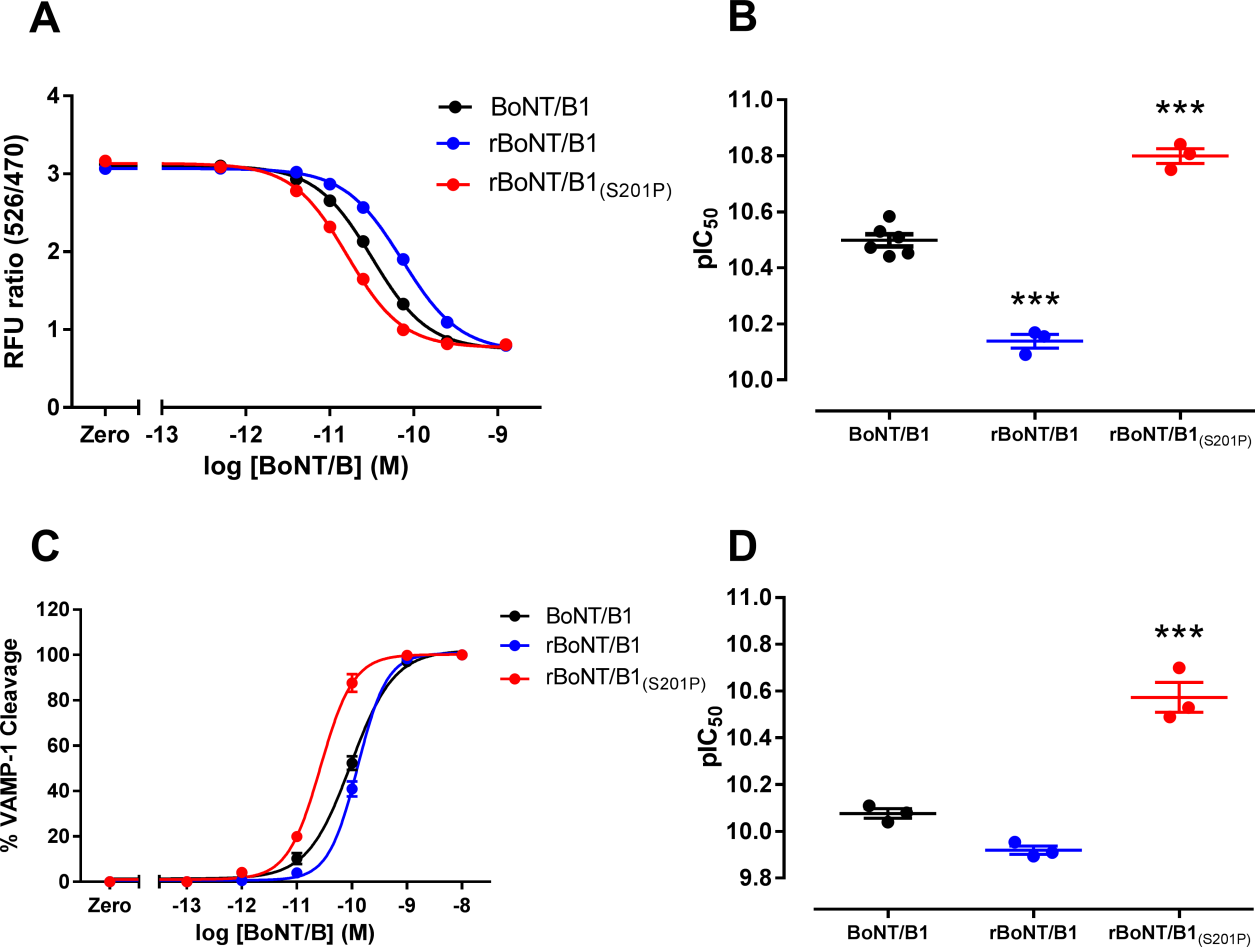
BoTest cell-free light chain activity assay. (A) BoNT/B1, rBoNT/B1 or rBoNT/B1_(S201P)_ (0.5 pM—1.25 nM) were incubated with 200 nM BoTest Reporter (VAMP-2 [33–94] flanked by N-terminal cyan fluorescent protein [CFP] and C-terminal yellow fluorescent protein [YFP]) for 18 hours at 30°C. Fluorescence intensity at 526 nm (YFP) and 470 nm (CFP) was determined using a BioTek Synergy HT plate reader. A diminishing 526/470 nm relative fluorescent unit (RFU) ratio indicates substrate cleavage. Data were fitted to a four-parameter equation. (B) The concentration of each toxin required for 50% maximum inhibition of 526/470 RFU ratio (pIC_50_) was determined from the fitted curves. Data represent the mean ± s.e.m. of *n* = 6 (BoNT/B1) or *n* = 3 (rBoNT/B1 and rBoNT/B1_(S201P)_) independent experiments, each performed in duplicate. (C) BoNT/B1, rBoNT/B1 or rBoNT/B1_(S201P)_ (0.1 pM—10 nM) were incubated with 2 μM VAMP-1 (2–96)-GFP at 37°C for 24 hr. Reactions were terminated by addition of NuPAGE LDS buffer, 1 mM DTT and separated by SDS-PAGE on 12% Bis-tris gels. Gels were stained and densitometry performed to determine the percentage substrate cleavage of each sample. Data were fitted to a four-parameter equation. (D) The concentration of each toxin required for 50% maximum substrate cleavage (pEC_50_) was determined from the fitted curves. Data represent the mean ± s.e.m. of *n* = 3 independent experiments, each performed in triplicate.Significant differences in potency are denoted by **(P<0.01) and ***(P<0.001, one-way ANOVA, Dunnett’s *post-hoc* test).

The light chain activities of BoNT/B1, rBoNT/B1 and rBoNT/B1_(S201P)_ were assessed in the BoTest cell-free assay by their ability to cleave a fluorescently labelled VAMP-2 substrate. BoNT/B1 was used in each assay as a reference toxin and the potencies of rBoNT/B1 and rBoNT/B1_(S201P)_ are expressed both in terms of absolute potency and % potency relative to BoNT/B. The potency (pIC_50_) of rBoNT/B1_(S201P)_ (10.80 ± 0.03, *n* = 3) was significantly (P<0.001) greater than the pIC_50_ of BoNT/B1 (10.50 ± 0.02, *n* = 6), with a relative potency of 187.7 ± 14.7% (Fig 2A and 2B and Table 1). In contrast, the potency of rBoNT/B1 (10.14 ± 0.02, *n* = 3) was significantly (P<0.001) lower than BoNT/B1 with a relative potency of 47.4 ± 4.5% (Fig 2A and 2B and Table 1).

**Table 1:** Summary data.

Model	BoNT/B1		rBoNT/B1		rBoNT/B1_(S201P)_	
**Light chain protease activity**						
VAMP-1 cleavage	pIC_50_:10.08 ± 0.02	Potency relative to BoNT/B1:100%	pIC_50_:9.92 ± 0.02	Potency relative to BoNT/B1:70.1 ± 6.0%	pIC_50_:10.57 ± 0.06***	Potency relative to BoNT/B1:324.7 ± 64.0%
	*n* = 3		*n* = 3		*n* = 3	
VAMP-2 cleavage:BoTest	pIC_50_:10.50 ± 0.02	Potency relative to BoNT/B1:100%	pIC_50_:10.14 ± 0.02***	Potency relative to BoNT/B1:47.4 ± 4.5%	pIC_50_:10.80 ± 0.03***	Potency relative to BoNT/B1:187.7 ± 14.7%
	*n* = 6		*n* = 3		*n* = 3	
**Activity in *in vitro* cell-based assays**						
Rat spinal cord neuronal culture: [^3^H]-glycine release	pIC_50_:11.57 ± 0.08	Max. Inhibition:82.8 ± 0.9%	pIC_50_:11.63 ± 0.12	Max. Inhibition:82.4 ± 0.7%	pIC_50_:11.52 ± 0.15	Max. Inhibition:83.6 ± 1.1%
	*n* = 7		*n* = 4		*n* = 4	
Rat cortical neuronal culture: glutamate release 5 hrs	Not determined		pIC_50_:9.03 ± 0.11	Max. Inhibition:93.6 ± 3.0%	pIC_50_:9.10 ± 0.10	Max. Inhibition:94.8 ± 1.8%
			*n* = 3		*n* = 3	
Rat cortical neuronal culture: glutamate release 24 hrs	pIC_50_:10.85 ± 0.08	Max. Inhibition:97.5 ± 3.4%	pIC_50_:10.59 ± 0.05	Max. Inhibition:96.1 ± 3.2%	pIC_50_:10.53 ± 0.07*	Max. Inhibition:98.8 ± 1.2%
	*n* = 4		*n* = 4		*n* = 4	
Rat cortical neuronal culture: Spontaneous synaptic activity	pIC_50_: 10.31 ± 0.04		pIC_50_: 9.91 ± 0.09*		pIC_50_: 9.91 ± 0.09**	
	*n* = 3		*n* = 3		*n* = 3	
**Activity in *ex vivo* tissue assays**						
CD1 mice:Hemi-diaphragm	t_10_ : 31.2 ± 1.2 min	t_50_: 47.1 ± 1.1 min	t_10_ : 29.7 ± 0.5 min	t_50_: 44.9 ± 1.2 min	t_10_ : 33.3 ± 1.9 min	t_50_: 52.9 ± 1.9 min*
	*n* = 4		*n* = 3		*n* = 3	
C57BL/6N mice:Hemi-diaphragm	t_10_ : 35.6 ± 0.6 min	t_50_: 52.6 ± 1.3 min	t_10_ : 35.8 ± 0.9 min	t_50_: 57.5 ± 2.5 min	t_10_ : 33.6 ± 2.1 min	t_50_: 54.8 ± 3.2 min
	*n* = 4		*n* = 4		*n* = 4	
C57BL/6N mice:Bladder Strip	t_10_ : 31.3 ± 2.8 min	t_50_: 58.4 ± 1.9 min	t_10_ : 26.9 ± 2.7 min	t_50_: 48.6 ± 3.3 min	t_10_ : 22.5 ± 1.6 min	t_50_: 51.2 ± 5.2 min
	*n* = 3		*n* = 3		*n* = 3	
**Activity *in vivo***						
C57BL/6N mice:Digit Abduction Score (DAS)	ED_50_:1.0 pg/mouse (95% confidence intervals 0.80 to 1.20 pg/mouse)	DAS 4:3.4 pg/mouse	ED_50_:1.3 pg/mouse(95% confidence intervals 0.95 to 1.65 pg/mouse)	DAS 4:3.5 pg/mouse	ED_50_:1.3 pg/mouse (95% confidence intervals 1.07 to 1.62 pg/mouse)	DAS 4:5.2 pg/mouse
C57BL/6N mice:Safety	BW-10%:3.6 pg/mouse	MTI:3.6	BW-10%:2.3 pg/mouse	MTI:1.8	BW-10%:3.0 pg/mouse	MTI:2.3

Significant differences in potency of rBoNT/B1 and rBoNT/B1_(S201P)_ compared to BoNT/B1 are denoted by *P<0.05, **P<0.01 and ***P<0.001 (One-way ANOVA, Dunnett’s *post-hoc* test).

We also tested the ability of BoNT/B1, rBoNT/B1 and rBoNT/B1_(S201P)_ to cleave the VAMP-1 isoform in cell-free conditions (Fig 2C and 2D and Table 1). The pEC_50_ of rBoNT/B1_(S201P)_ (10.57 ± 0.06, *n* = 3) was significantly (P<0.001) greater than the pEC_50_ of both BoNT/B1 (10.08 ± 0.02, *n* = 3) and rBoNT/B1 (9.92 ± 0.02, *n* = 3) (Fig 2C and 2D and Table 1).

### *In vitro* potency of BoNT/B1, rBoNT/B1 and rBoNT/B1_(S201P)_ in rat primary cortical and spinal cord neurons

The *in vitro* potencies of BoNT/B1, rBoNT/B1 and rBoNT/B1_(S201P)_ were evaluated in cell-based neurotransmitter release assays using both rat primary spinal cord and cortical neurons, to allow comparison of the effect of the toxins on release of inhibitory and excitatory neurotransmitters.

**Fig 3:**
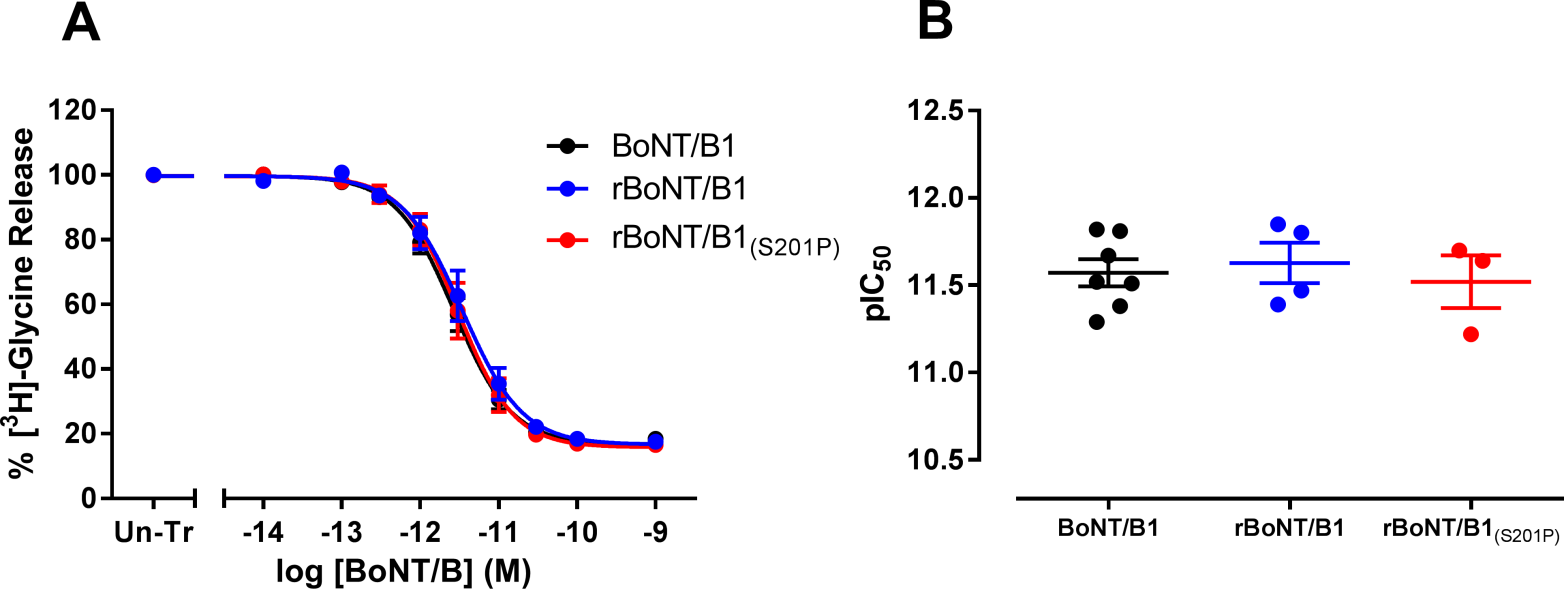
Inhibition of [^3^H]-glycine release from rat spinal cord neurons. (A) Primary rat spinal cord neurons were incubated for 24 hours with BoNT/B1, rBoNT/B1 or rBoNT/B1_(S201P)_ (10 fM—1 nM). Cells were loaded with [^3^H]-glycine for 60 minutes in HEPES-buffered saline (HBS) before washing and assessment of basal and stimulated [^3^H]-glycine release for 5 minute periods using HBS solutions containing 3 mM or 60 mM potassium, respectively. Radioactivity associated with cell superfusates was determined by liquid scintillation counting. Data were normalised to the [^3^H]-glycine release obtained from un-treated cells (Un-Tr) and fitted to a four-parameter equation. (B) The concentration of each toxin required for 50% maximal inhibition of [^3^H]-glycine release (pIC_50_) was determined from the fitted curves. Data represent the mean ± s.e.m. of *n* = 7 (BoNT/B1), *n* = 4 (rBoNT/B1) or *n* = 3 (rBoNT/B1_(S201P)_) independent experiments, each performed in triplicate. No significant differences in potency were observed (one-way ANOVA).

**Fig 4:**
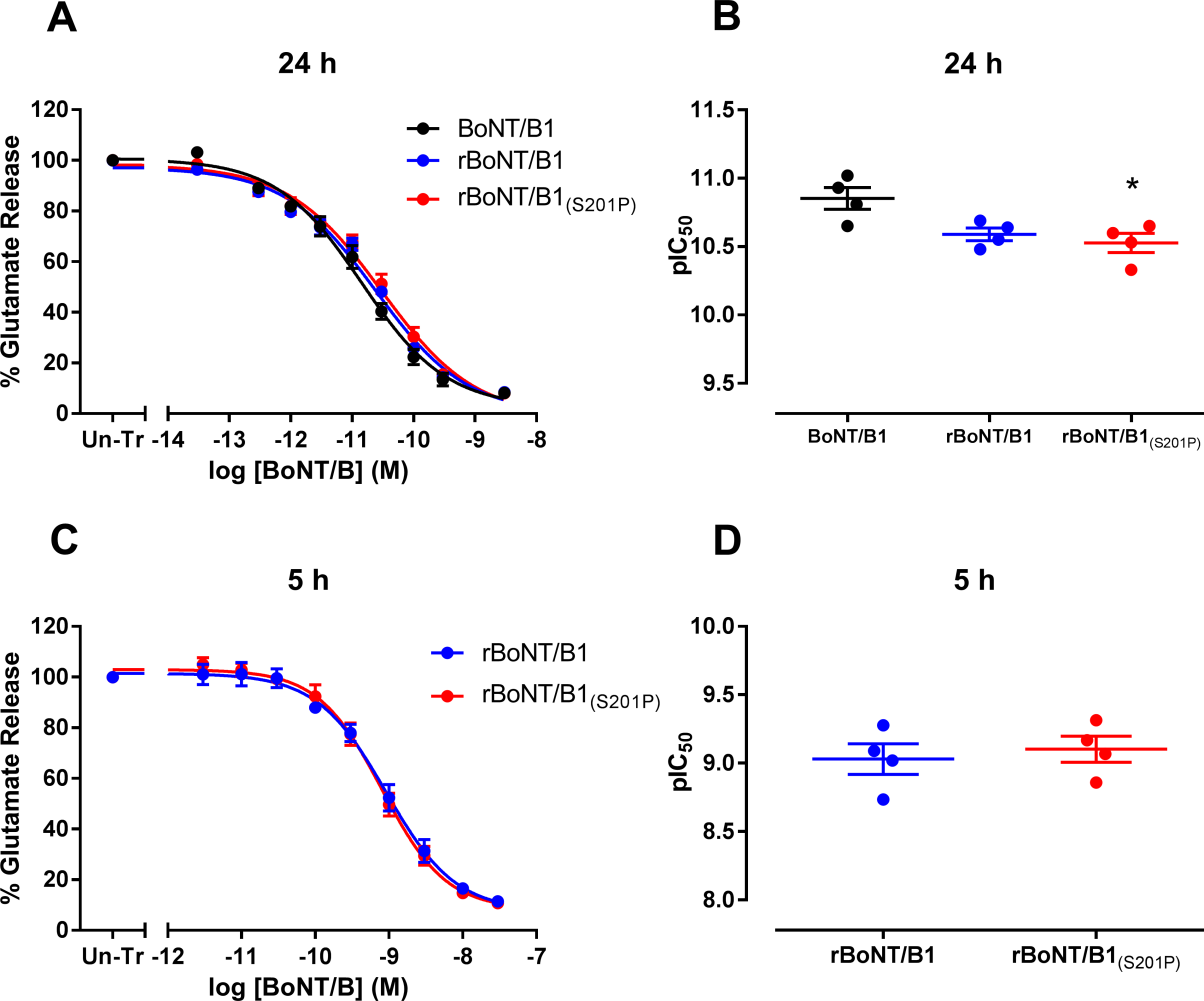
Inhibition of glutamate release from rat cortical neurons. Primary rat cortical neurons were incubated for (A) 24 hours (30 fM—3 nM) or (B) 5 hours (3 pM—30 nM) with rBoNT/B1 or rBoNT/B1_(S201P)_. Cells were pre-incubated with assay medium containing TFB-TBOA (excitatory amino acid transporter inhibitor) for 30 minutes before washing and assessment of basal and stimulated glutamate release for 5 minute periods using assay medium containing 5 mM or 60 mM potassium, respectively. Glutamate content of cell superfusates was determined using an Amplex Red fluorometric assay. Data were normalised to the glutamate release obtained from un-treated cells (Un-Tr) and fitted to a four-parameter equation. The concentration of each toxin required for 50% maximal inhibition (pIC_50_) of glutamate release was determined from the fitted curves for (C) 24 hours and (D) 5 hours toxin exposure. Data represent the mean ± s.e.m. of *n* = 4 independent experiments, each performed in triplicate. Significant difference in toxin potency compared to BoNT/B1 is denoted by *(P<0.05 (one-way ANOVA, Dunnett’s *post-hoc* test).

In rat primary spinal cord neurons, BoNT/B1 (pIC_50_ = 11.57 ± 0.08, *n* = 7), rBoNT/B1 (pIC_50_ = 11.63 ± 0.12, *n* = 4) and rBoNT/B1_(S201P)_ (pIC_50_ = 11.52 ± 0.15, *n* = 3) were equipotent in inhibiting the release of pre-loaded [^3^H]-glycine following a 24 hour toxin treatment (one-way ANOVA; Fig 3 and Table 1). The ability of the toxins to inhibit the release of endogenous glutamate from rat primary cortical neurones was also assessed. rBoNT/B1_(S201P)_ (pIC_50_ = 10.53 ± 0.07, *n* = 4) was found to be equipotent with rBoNT/B1 (pIC_50_ = 10.59 ± 0.05, *n* = 4), but significantly less potent than BoNT/B1 (pIC_50_ = 10.85 ± 0.07, *n* = 4) (P<0.05, one-way ANOVA, Dunnett’s *post-hoc* test, Fig 4A and 4C and Table 1).

We assessed whether there was a difference between rBoNT/B1 and rBoNT/B1_(S201P)_ in the onset of action, by measuring the release of endogenous glutamate from rat primary cortical neurons after 5 hours of toxin treatment (Fig 4B and 4D). As expected for a shorter toxin incubation time, the activity of rBoNT/B1 (pIC_50_ = 9.03 ± 0.11, *n* = 4) was lower as compared to 24 h incubation. This was also the case for rBoNT/B1_(S201P)_ (pIC_50_ = 9.10 ± 0.10, *n* = 4). However, also with a 5 hour toxin incubation there was no significant difference between rBoNT/B1 and rBoNT/B1_(S201P)_ (un-paired t-test, P = 0.641; Table 1).

**Fig 5:**
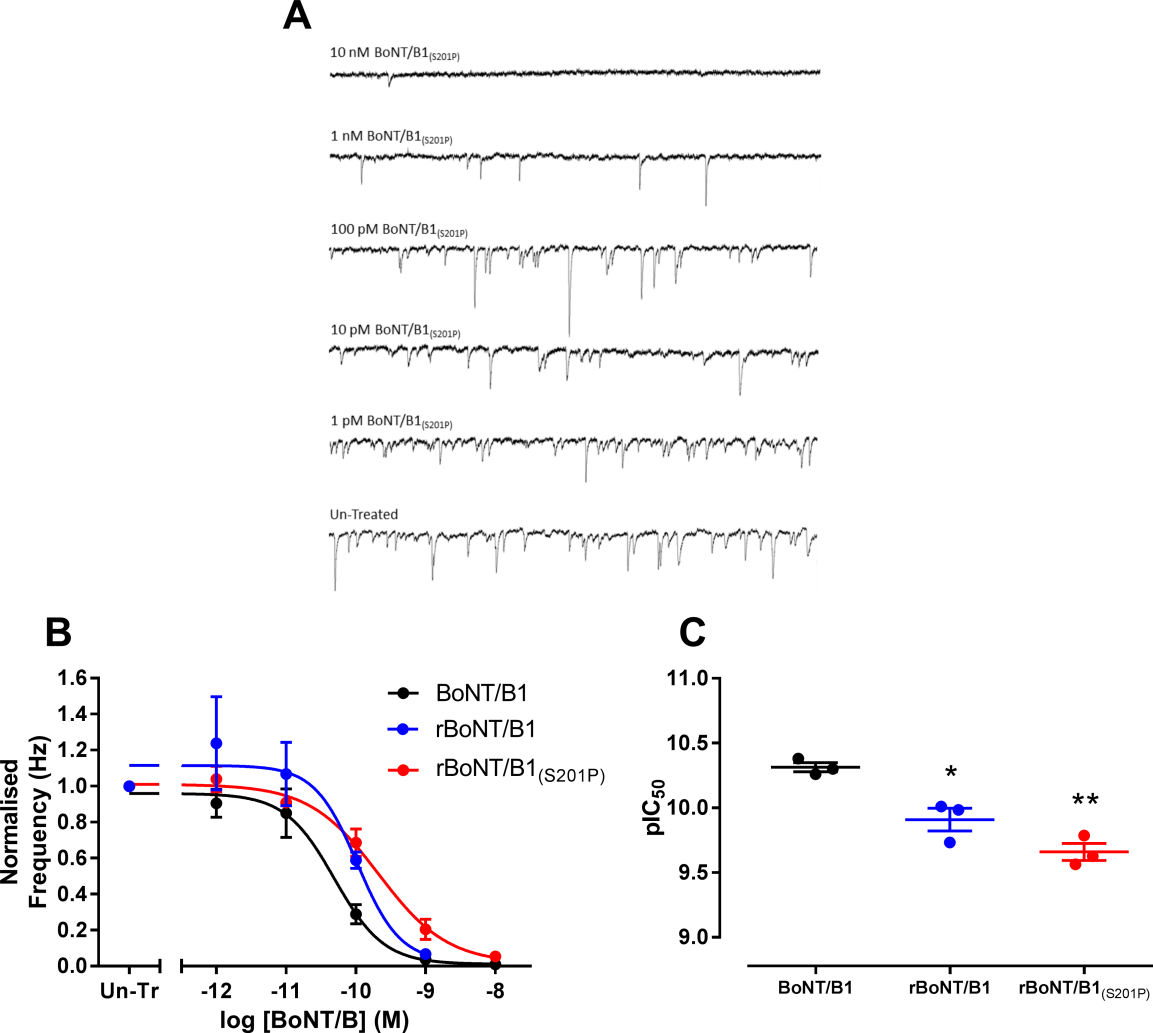
Inhibition of mEPSCs from rat cortical neurons. (A) Primary rat cortical neurons were incubated for 24 hours with rBoNT/B1 or rBoNT/B1_(S201P)_ (1 pM—10 nM). Whole cell recordings were made a holding potential of -70 mV from cells perfused with artificial cerebrospinal fluid containing tetrodotoxin to block action potentials and GABA receptor antagonists to block inhibitory currents. The frequency of miniature excitatory post-synaptic currents (mEPSC) was determined from 5–6 different cells for each toxin concentration. (B) Data were normalised to the mEPSC frequency recorded from un-treated cells (Un-Tr) and fitted to a four-parameter equation. (C) The concentration of each toxin required for 50% maximal inhibition (pIC_50_) of mEPSC frequency was determined from the fitted curves. Data represent the mean ± s.e.m. of *n* = 3 independent experiments. Significant differences in potency compared to BoNT/B1 are denoted by *(P<0.05) and **(P<0.01) (one-way ANOVA, Dunnett’s *post-hoc* test).

Rat cortical neuron cultures were also tested in patch-clamp electrophysiology assays to measure the inhibition of spontaneous neural network activity in cultures exposed to different concentrations of toxin. In this assay, BoNT/B1 (pIC_50_ = 10.31 ± 0.04, *n* = 3) was significantly more potent than both rBoNT/B1 (pIC_50_ = 9.91 ± 0.09, *n* = 3, P<0.05) and rBoNT/B1_(S201P)_ (pIC_50_ = 9.66 ± 0.07, *n* = 3, P<0.01 one-way ANOVA, Dunnett’s *post-hoc* test; Fig 5 and Table 1).

### *Ex vivo* potency of BoNT/B1, rBoNT/B1 and rBoNT/B1_(S201P)_ in mouse phrenic nerve hemi-diaphragm and bladder strip organ bath assays

**Fig 6:**
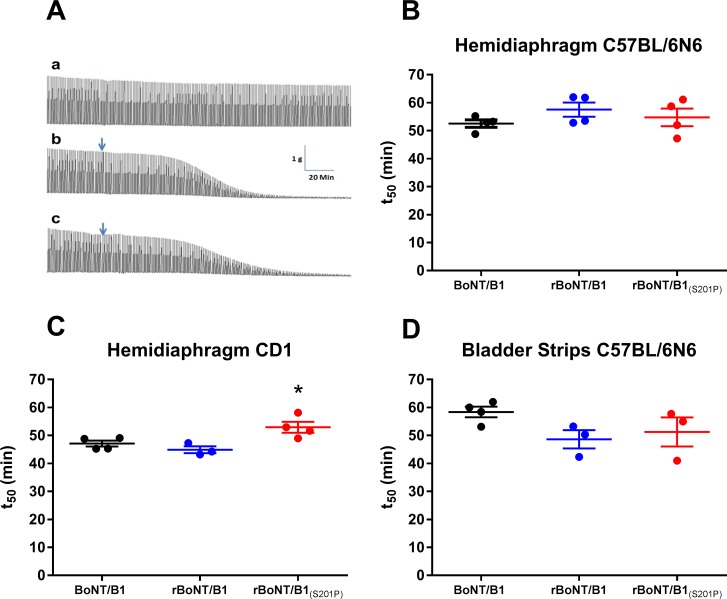
Mouse phrenic nerve hemi diaphragm and bladder strip *ex vivo* assays. (A) The left phrenic nerve and hemi-diaphragm were removed from either C57BL/6N or CD1 mice and suspended in organ baths in Krebs-Henseleit buffer (KHB). The phrenic nerve was stimulated at a frequency of 1 Hz, 20 µs duration and the contractile force of the hemi-diaphragm muscle was recorded. Following tissue stabilisation, rBoNT/B1 (b) or rBoNT/B1_(S201P)_ (c) were added to the bath at 10 pM final concentration (denoted by the arrows, traces are from C57BL/6N mice). The top trace (a) shows a control trace in KHB. (B) Data were normalised to the contractile strength prior to toxin addition and fitted to a four-parameter equation. The time required to decrease the contractile strength by 50% (t_50_) was determined from the fitted curves for hemi-diaphragm preparations from C57BL/6N mice (B) and CD1 mice (C). (D) Strips of detrusor muscle were dissected from the bladders of C57BL/6N mice and suspended in organ baths in KHB. Electrical field stimulation (EFS) at 10 Hz, 0.3 ms pulse duration was used to evoke contractions of the muscle and the contractile force was recorded. Following tissue stabilisation, rBoNT/B1 or rBoNT/B1_(S201P)_ were added to the bath at 1 nM final concentration. Data were normalised to the contractile strength prior to toxin addition and fitted to a four-parameter equation. The time required to decrease the contractile strength by 50% (t_50_) was determined from the fitted curves. All data represent the mean ± s.e.m. of *n* = 4 (hemi-diaphragm, except rBoNT/B, CD1 mice, *n* = 3) or *n* = 3 (bladder strip) independent tissue preparations for each toxin. Significant differences in potency compared to BoNT/B1 are denoted by *(P<0.05) (one-way ANOVA, Dunnett’s *post-hoc* test).

To test whether rBoNT/B1_(S201P)_ would show improved activity in a multi cellular organ bath system, we first used the mouse phrenic nerve hemi-diaphragm preparation. In this assay, diaphragm contractions rely only on acetylcholine release which stimulates nicotinic receptors at the post-synaptic level [21]. When using hemi-diaphragm preparations obtained from C57BL/6N mice, the addition of 10 pM BoNT/B1, rBoNT/B1 or rBoNT/B1_(S201P)_ to the bath caused a gradual decrease of the contractile force of the preparation that finally resulted in full paralysis of the muscle. The time to achieve 50% paralysis of the diaphragm (t_50_) was 52.6 ± 1.3 min (*n* = 4), 57.5 ± 2.5 min (*n* = 4) and 54.8 ± 3.2 min (*n* = 4) for BoNT/B1, rBoNT/B1 and rBoNT/B_(S201P)_, respectively. The onset of action in the model, as measured by the t_10_ values was 35.6 ± 0.6 min (*n = 4*) for BoNT/B1, 35.8 ± 0.9 minutes (*n* = 4) for rBoNT/B1 and 33.6 ± 2.1 minutes (*n* = 4) for rBoNT/B1_(S201P)_. There were no significant differences between the t_50_ or the t_10_ values of the three toxins (One-way ANOVA, Fig 6A and 6B and Table 1). In hemi-diaphragm preparations obtained from CD1 mice, the t_50_ of rBoNT/B1 (44.9 ± 1.2 minutes, *n* = 3) was equipotent to BoNT/B1 (47.1 ± 1.1 minutes, *n* = 4), but significantly lower than the t50 of rBoNT/B1_(S201P)_ (52.9 ± 1.9 minutes, *n* = 4; one-way ANOVA, Dunnett’s *post-hoc* test, Fig 6C and Table 1). However, there was no significant difference in the onset of action in CD1 mice, as measured by the t_10_ values, (BoNT/B1 = 31.2 ± 1.2 minutes (*n* = 4); rBoNT/B1 = 29.7 ± 0.5 minutes (*n* = 3); rBoNT/B1_(S201P)_ = 33.3 ± 1.9 minutes (*n* = 4)), (one-way ANOVA, Fig 6C and Table 1).

We also tested the potency of the toxins in mouse bladder strip preparations obtained from C57BL/6N mice. This preparation is a smooth muscle preparation and we have previously shown that under our experimental conditions approximately 50% of the signal in this preparation is due to purinergic neurotransmission, whereas the remainder is due to release of acetylcholine stimulating muscarinic receptors post-synaptically [18, 22]. BoNT/B1, rBoNT/B1 and rBoNT/B1_(S201P)_ induced a gradual decrease in the amplitude of contractions, with t_50_ values of 58.4 ± 1.9 minutes (*n* = 3) for BoNT/B1, 48.6 ± 3.3 minutes (*n* = 3) for rBoNT/B1 and 51.2 ± 5.2 minutes (*n* = 3) for rBoNT/B1_(S201P)_ (Fig 6D). The onset of action in the model, as measured by the t_10_ values were 31.3 ± 2.8 minutes (*n* = 3) for BoNT/B1, 26.9 ± 2.7 minutes (*n* = 3) for rBoNT/B1 and 22.5 ± 1.6 minutes (*n* = 3) for rBoNT/B1_(S201P)_. There were no significant differences in either the t_10_ or the t_50_ between the toxins (one-way ANOVA, Table 1).

### In vivo activity of rBoNT/B1 and rBoNT/B1_(S201P)_ in mouse Digit Abduction Score (DAS) assay

**Fig 7:**
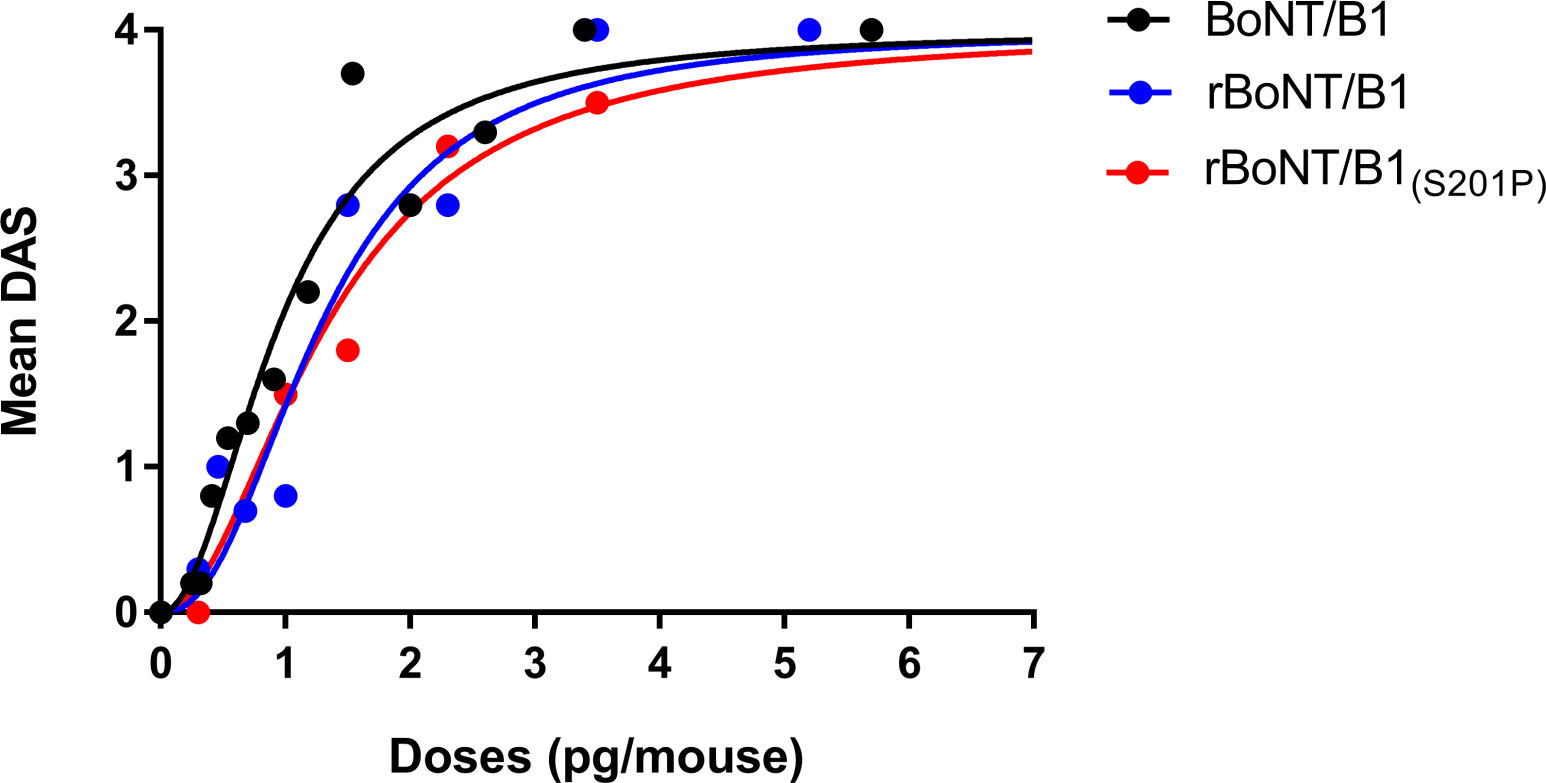
In vivo Digit Abduction Score (DAS) assay. BoNT/B1 (0.25 to 5.7 pg/mouse), rBoNT/B1 or rBoNT/B1_(S201P)_ (0.3 to 5.2 pg/mouse) were injected into the gastrocnemius-soleus muscle complex of the hind paw of C57BL/6N mice (6 animals for each dose). DAS was scored daily during the first four days after injection and the highest average DAS score per dose group during this period were plotted and fitted with the nonlinear equation, as given in Material and Methods. ED_50_ values and lower and upper 95% confidence intervals were derived from the fit.

Injection of rBoNT/B1 or rBoNT/B1_(S201P)_ into the gastrocnemius-soleus muscle complex of the right hind paw increased the DAS values in a dose-related manner (Fig 7, Table 1). The potency of the toxins, defined as the ED_50_, (the dose calculated to induce a mean DAS of 2) was 1.3 pg/mouse for both rBoNT/B1 and rBoNT/B1_(S201P)_ and 1.0 pg/mouse for BoNT/B1_._ The maximum DAS of 4 was attained with doses of 3.4 pg/mouse for BoNT/B1, 3.5 and 5.2 pg/mouse for rBoNT/B1 and rBoNT/B1_(S201P)_, respectively (Fig 7 and Table 1).

Safety parameters for each toxin were assessed as part of the DAS assay. The doses at which the adverse side effect of a 10% decrease in body weight became first apparent were 3.6, 2.3, and 3.0 pg/mouse, for BoNT/B1, rBoNT/B1 and rBoNT/B1_(S201P)_, respectively. The maximal tolerated index (MTI), defined at the dose causing 10% BW loss divided by the calculated ED_50_, was used as an indication of the safety window of the toxin, with higher numbers indicating increased safety. The MTI for the three toxins were 3.6 for BoNT/B1, 1.8 for rBoNT/B1 and 2.3 for rBoNT/B1_(S201P)_.

## Discussion

In the present study we have produced a full-length BoNT/B1 toxin incorporating a S201P mutation (rBoNT/B1_(S201P)_) and characterised the activity of this modified botulinum neurotoxin in a range of *in vitro*, *ex vivo* and *in vivo* assays. In the context of the interaction between LC/B and its VAMP-2 substrate, serine 201 is one of five residues located in the S1′ binding pocket of the enzyme. The LC of TeNT (LC/T), which cleaves VAMP-2 at the same scissile bond as LC/B, contains a similar S1′ binding pocket. Substitutions of structurally aligned residues within the S1′ pockets of LC/B and LC/T revealed proline 205 in LC/T to be more optimal than the corresponding serine 201 within LC/B [15]. Exchange of S201 for a proline augments the catalytic activity of the isolated BoNT/B LC domain in biochemical assays by increasing the substrate turnover (*K*_cat_) [14, 15]. We find that although incorporation of this mutation into the full-length active toxin rBoNT/B1_(S201P)_ does indeed result in a toxin with higher catalytic activity than BoNT/B1 and rBoNT/B1 cell-free assays of VAMP-2 or VAMP-1 cleavage, our data in a comprehensive range of *in vitro*, *ex vivo* and *in vivo* assays, show that this does not translate into a higher potency in complex biological systems. The potency of BoNT/B1_(S201P)_ was not significantly greater than the wild type BoNT/B1 toxins in any of the *in vitro*, *ex vivo* or *in vivo* assays. Our results suggest that the cleavage rate of the SNARE substrate is not, at least in the case of BoNT/B1, determining the overall potency of the toxin, nor does it appear to be the rate-limiting step in the onset of action.

Before this study, the S201P mutation had only been verified in cell-free cleavage assays using VAMP-2 as substrate [15]. Indeed, most studies examining the *in vitro* activity of BoNT/B focus on the VAMP-2 isoform. This is perhaps largely due to the availability of cell models, with central nervous system (CNS)-derived primary neuronal models being most commonly used. However, recent evidence shows that the major isoform of VAMP at both rodent and human motor nerve terminals is VAMP-1, while VAMP-2 is most abundant in central neurons [23, 24]. VAMP-1 and VAMP-2 isoforms share nearly 90% sequence identity and a previous cell-free study reported that the ability of LC/B to cleave VAMP-1 and VAMP-2 is very similar [25]. In the present study, BoNT/B1_(S201P)_ demonstrated a superior ability to cleave both VAMP-1 and VAMP-2 compared to either native or recombinant sources of wild type BoNT/B1. The enhanced catalytic activity of BoNT/B1_(S201P)_ is, therefore, relevant in assays where VAMP-1 (*ex vivo* and *in vivo*) or VAMP-2 (*in vitro*) is likely the most relevant isoform.Processes other than the catalytic activity of the LC may determine the potency and time to onset of action of BoNT/B in physiological systems. A key event in the overall intoxication process is the binding of BoNT/B to the neuronal membrane, achieved via binding to the gangliosides GD1a and GT1b, followed by binding to its protein receptor, synaptotagmins [26–30]. Indeed, modifications in the ganglioside binding domain [29, 31] as well as the synaptotagmin binding domain of BoNT/B [32] result in increased activity of the mutated toxins in physiological systems. Thus, we have recently shown that a full-length rBoNT/B1 toxin containing a double mutation shown to enhance affinity to human synaptotagmin II also exhibited ~10-fold higher potency in blocking neurotransmission compared to unmodified rBoNT/B in cultured neurons that express human synaptotagmin II [32].

Another of the critical, but least well understood, steps of botulinum neurotoxin intoxication is the translocation process [33, 34]. It could be that modifications influencing the speed of translocation manifest themselves as changes in the overall efficacy of botulinum neurotoxins. Furthermore, for BoNT/B it has been proposed that translocation of the LC requires oligomerisation of several BoNT/B translocation domains [35], which could further impact on the speed of this process. Indeed, translocation of the BoNT/B light chain into the cytoplasm is slow, as it is still sensitive to inhibition of vesicle acidification by Concanamycin A up to 6 hours after toxin treatment [36]. The kinetics of BoNT/B translocation channel development have not been studied on a microscopic scale with single channel recordings, but for a truncated BoNT/A containing only the LC and translocation domain regions, the speed of this process requires minutes to occur, with time constants of 130 seconds at pH 5 and 190 seconds at pH 6 [37]. Indeed, modifications in BoNT/B that affect the speed of the translocation process have been found to result in increased activity of the neurotoxin in physiological systems [38]. In this paper, double and triple combinations of neutralizing mutations of three carboxylatic residues did increase speed of translocation of the light chain, as well as overall efficacy of the corresponding mutated toxins in a mouse hemi-diaphragm assay.

Linked to the translocation process is release of the LC into the cytoplasm through reduction of the interchain disulfide bond. The NADPH-thioredoxin reductase–thioredoxin system is critically involved in this process for BoNT/B and other BoNT serotypes [34, 39]. Interestingly, the reduction of the interchain disulfide bond is the rate-limiting step in the intoxication process of diphtheria toxin [40].

These findings suggest that several steps in the intoxication process of BoNT/B can be rate-limiting at a macroscopic level. Thus, the augmentation of the protein receptor affinity may have effects at the multicellular level (increased number of cells with vesicles containing a neurotoxin molecule), the single cell level (increased ratio of vesicles containing a neurotoxin over the total number of vesicles, and/or increased number of neurotoxin molecules per vesicle), or both. In contrast, the modifications reported by Pirazzini *et al* [38] will result in a greater number of LCs being transported into the cytoplasm per unit time. Given the slowness of this process, it is likely that the data by Pirazzini *et al* [38] also reflect the true rate-limiting step on a microscopic level. However, because the read-outs used in the Pirazzini study also require the cytoplasmic release of the translocated LC, the rate-limiting step may not necessarily be the translocation process itself. In any case, it would be interesting to explore whether modifications that increase protein receptor affinity combined with modifications that increased speed of cytoplasmic delivery of the LC would further augment the onset of action and potency of such a modified BoNT/B toxin.

While our results indicate that increasing the catalytic activity of the BoNT/B LC by the S201P mutation [14, 15] does not result in higher potency in complex biological systems, it is not known whether our findings can be generalized to other BoNTs. In fact, despite significant advances in understanding the molecular details of SNARE substrate recognition and BoNT specificity [11–15, 41–44], only a few modifications have been identified that result in increased catalytic activity of the original SNARE substrate. Besides the modifications described for BoNT/B [14, 15], we are only aware of two other studies describing function-enhancing mutations in the LC of other BoNT serotypes–both involving BoNT/A. In one study, the single amino acid exchange of K165L in the BoNT/A LC was shown to increase catalytic activity approximately 4-fold [45]. The second reports a modest 1.5-fold increase in SNAP-25 cleavage by LC/A_(K340R)_ [46]. Since full-length active toxins containing these mutations have not been produced, it is not possible to compare the effects of incorporation in complex biological systems to our findings.

Our data suggest that increasing the catalytic activity of BoNT/B LC is not a suitable way to lower the required treatment dose of BoNT/B in the clinical setting. Thus, this strategy is not sufficient to overcome some of the drawbacks associated with the marketed BoNT/B product, such as the higher incidence of adverse effects [4] and higher incidence of immune reactions [7]. In order to achieve the goal of improving BoNT/B efficacy, other engineering strategies should also be considered. Our recent finding that a modified recombinant BoNT/B1 toxin with enhanced affinity to its human synaptotagmin II receptor blocks neurotransmission with higher potency in cellular assays as compared to unmodified BoNT/B may point to one such alternative approach [32].
